# Prevalence of Intestinal Parasites in Food Handlers of Bandar Abbas, Southern Iran

**Published:** 2018-01

**Authors:** Mehrgan HEYDARI-HENGAMI, Yaghoob HAMEDI, Majid NAJAFI-ASL, Khojasteh SHARIFI-SARASIABI

**Affiliations:** Infectious and Tropical Diseases Research Center, Hormozgan Health Institute, Hormozgan University of Medical Sciences, Bandar Abbas, Iran

**Keywords:** Prevalence, Intestinal parasites, Food handlers, Iran

## Abstract

**Background:**

Food handlers play a significant role in the dissemination and transmission of enteropathogenic microorganisms, including intestinal parasites. The aim of this study was to determine the prevalence of intestinal parasites in food handlers of Bandar Abbas, southern Iran.

**Methods:**

In this cross-sectional study, 800 stool samples were randomly collected in a period of 6 months from Jun to Nov 2015. The diagnosis was made on direct wet mount, formalin-ether concentration, Ziehl-Neelsen, and Trichrome stained slides.

**Results:**

34.9% of participants were positive for stool parasites. The most infected individuals were seen in the workers of bakeries 54.3%, factories 41.1% fast foods 35.7%, supermarkets 34.7%, restaurants 33.9%, offices 29.8%, butchers 27.3% and coffee shops 26.7% respectively (*P*<0.05). The intestinal parasites were *Blastocystis hominis* 24.3%, *Entamoeba coli* 8%, *Giardia lamblia* 6.8% and *Dientamoeba fragilis* 4.3% respectively. Only two infections by *Hymenolepis nana* (0.3%) and one by *Enterobius vermicularis* (0.1%) were detected in this study. Living in the workplace and direct contact with the raw foodstuff affected the prevalence of intestinal parasites (*P*<0.05).

**Conclusion:**

The use of concentration methods for the detection of intestinal parasites as well as continuous and effective education in short-term courses to promote hygienic behavior is suggested.

## Introduction

Transmission of intestinal parasites, either directly or indirectly through contaminated food, water or hands represent importance of fecal-oral or human-to-human transmission (1). Contaminated food which causes diseases considered a major threat to public health as the main source targeting illness and death and also as a significant impediment to socio-economic development worldwide (2). Some jobs may facilitate the transmission of parasites such as those deals with food. Food handlers with a low level of personal hygiene could be potential sources of parasitic worms, protozoa, as well as intestinal pathogenic bacteria (3). The intestinal pathogens excreting through the feces cause the contamination of the fingers and consequently the foods while processing which accordingly infects the healthy individuals (4). Compared with the rest part of the hand, fingernails, due to the surviving of micro-organisms, are filthier than other parts which are more difficult to keep them clean (5). Furthermore, if the infected people with intestinal parasites, treated incompletely, they can act as a carrier of the enteric parasites to healthy individuals (6). Since parasitic infections often tend to be chronic, asymptomatic individuals who work as food handlers may become potential sources of contamination and dissemination of several enteric pathogens (7).

According to National Food Safety Standards in Iran all food handlers should be referred to a health center medical diagnostic laboratory in order to check for intestinal parasitic infections prior to receiving their health certificate (1).

The prevalence of intestinal parasite among food handlers has been reported 19% in western Iran (6), 14.7% in Kerman (8), and 10.4% in Shiraz (4) respectively. A similar prevalence has been suggested 29% in northeastern India (9) and northwest Ethiopia (5).

The present study among the whole foodstuff handlers of Bandar Abbas has been carried out on a larger scale using complementary methods besides direct and formalin-ether techniques. We aimed to determine the prevalence of intestinal parasites in food handlers of Bandar Abbas, southern Iran, as well as its association with demographic characteristics.

## Methods

### Study population and sample collection

In this cross-sectional study, 800 stool samples were randomly collected from food handlers in Bandar Abbas and its suburbs, Hormozgan Province, Iran in a period of 6 months from Jun to Nov 2015. This city is located in southern Iran; a tropical region, attached to the Persian Gulf with high humidity (20%–100%) and warm climate (10). Bandar Abbas is situated on a flat area with an average altitude of 9 meters (30 ft.) above the sea level and the population of approximately 450000. Maximum temperature in summers can reach 49 °C (120 °F) while in winters the minimum temperature may drop to 5 °C (41 °F) (https://en.wikipedia.org/wiki/Bandar_Abbas).

Infectious and Tropical Diseases Research Center, HUMS, Bandar Abbas, Iran approved the study. The ethical code of the project (HUMS.REC.1394.153) obtained from the Ethics Committee of the HUMS. The objective of the study was explained to the participants to get informed written consent.

Data were collected using the questionnaire prepared on the basis of age, gender, place of living (rural or urban), educational level, health certificate (yes or no) and number of family members, number of colleagues, direct contact with raw foodstuff and living at workplace. All of the participants used drinking healthy water and used the toilet for defecation.

For each food handler, three fresh fecal specimens were collected in a clean stool cup every other day. Each container was labeled and immediately sent to the parasitology laboratory at the School of Medicine of Hormozgan University of Medical Sciences (HUMS). The diagnosis was made on direct wet mount, formalin-ether concentration, Ziehl-Neelsen, and Trichrome stained slides.

### Statistical analysis

The collected data were analyzed using SPSS software (version 20, Chicago, IL, USA). The prevalence of intestinal parasites evaluated with descriptive statistics, while the relationship between the variables and presence of intestinal parasites assessed by the chi-square test. The level of significance was 0.05.

## Results

The infection status was as follow; out of 800 individuals examined, 279 (34.9%) were infected with at least one intestinal parasite; including 218 (78.1%) males and 61 females (21.9%), ( Table 1–3). Of the total stools examined, 684 (85.5%) formed, 41 (5.1%) soft, 58 (7.3%) loose and 17 (2.1%) were diarrheal form. The most infection was seen on Jun 85 (31.7%) and the least on Nov, 13 (4.9%). The most infection rate was seen in the 20–29 age group (34.7%) but no statistically significant difference reported between the prevalence of intestinal parasites neither among each age group (*P*=0.152) nor genders (*P*=0.536) (Table 1).

**Table 1:** Relationship between prevalence of intestinal parasitic infections and certain assumed risk factors among 800 food handlers in Bandar Abbas, southern Iran, 2015

***Variables***	***Frequency (%)***	***Parasitic infection (%)***	***P value***
**Sex**			
Male	625 (78.1)	218 (78)	0.536
Female	175 (21.9)	61 (22)	
**Age group(yr)**			
<19	50 (6.2)	17 (34)	0.152
20–29	311 (38.9)	108 (34.7)	
30–39	254 (31.8)	87 (34.3)	
40–49	116 (14.5)	50 (43.1)	
>50	69 (8.6)	17 (24.6)	
**Place of living**			
Urban	666 (83.3)	234 (35.1)	0.406
Rural	134 (16.8)	45 (33.6)	
**Educational level**			
<Diploma	397 (49.6)	136 (34.3)	0.240
Diploma	299 (37.4)	113 (37.8)	
Academic degree	104 (13)	30 (28.8)	
**Number of family members**			
1–2	131 (16.4)	42 (32.1)	0.754
3–5	560 (70)	199 (35.5)	
≥6	109 (13.6)	38 (34.9)	
**Number of colleagues**			
1–3	282 (35.3)	94 (33.3)	0.582
4–9	268 (33.5)	100 (37.3)	
≥10	250 (31.3)	85 (34)	
**Direct Contact with raw food**			
Yes	721 (90.1)	259 (35.9)	0.038
No	79 (9.9)	20 (25.3)	
**Health certificate**			
Yes	538 (67.3)	189 (35.1)	0.446
No	262 (32.7)	90 (34/4)	
**Living at work place**			
Yes	149 (18.6)	96 (64.4)	0.000
No	183 (81.4)	183 (28.1)	

**Table 2:** Types and prevalence of intestinal parasites in stool samples among 800 food-handlers in Bandar Abbas, southern Iran, 2015

***Intestinal parasites***	***Number***	***Percent n=279***	***Percent n=800***
*B. hominis*	194	69.5	24.3
*E. coli*	64	22.9	8
*G. lamblia*	54	19.4	6.8
*D. fragilis*	34	12.2	4.3
*I. butschlii*	8	2.9	1
*E. histolytica/dispar*	7	2.2	0.9
*E. nana*	2	0.7	0.3
*E. vermicularis*	1	0.4	0.1
*H. nana*	2	0.7	0.3

**Table 3:** Prevalence of intestinal parasitic infections based on the number of parasites among 800 food handlers, in Bandar Abbas, southern Iran, 2015

***Intestinal Parasites***	***Frequency***	***Percent n=279***	***Percent n=800***
**Single infection**			
*G. lamblia*	26	9.3	3.3
*B. hominis*	125	44.8	15.6
*E. coli*	28	10	3.5
*E. histolytica/dispar*	3	1.1	0.4
*D. fragilis*	7	2.5	0.9
*H. nana*	2	0.7	0.3
*E. vermicularis*	1	0.4	0.1
**Double infections**			
*B. hominis+E. coli*	25	9	3.1
*B. hominis+G. lamblia*	13	4.7	1.6
*B. hominis+D. fragilis*	11	3.9	1.4
*B. hominis+C.mesnili*	4	1.4	0.5
*B. hominis+I. butschlii*	3	1.1	0.4
*B.hominis+E. histolytica/dispar*	3	1.1	0.4
*E. coli+G. lamblia*	5	1.8	0.6
*E. coli+I. butschlii*	2	0.7	0.3
*D. fragilis+E. nana*	1	0.4	0.1
*D. fragilis+I. butschlii*	1	0.4	0.1
*G. lamblia+C. mesnili*	3	1.1	0.4
**Triple infections**			
*B. hominis+E. coli+D. fragilis*	3	1.1	0.4
*B. hominis+D. fragilis+G. lamblia*	4	1.4	0.5
*B. hominis+C. mesnili +D. fragilis*	2	0.7	0.3
*B. hominis+E. coli+G. lamblia*	1	0.4	0.1
*G. lamblia+E. coli+C. mesnili*		0.4	0.1
*E.coli+I. butschlii+E. histolytica/dispar*	1	0.4	0.1
*G. lamblia+E. coli+D. fragilis*	1	0.4	0.1
**Quadruple infections**			
*B.hominis+E.nana+D.fragilis+I. butschlii*	1	0.4	0.1
Total infection	279	100	34.9

The highest prevalence of intestinal parasites was found in the bakery workers included delivery of bread, bread cooking and cashier (n=44, 54.3%) with the majority of *Blastocystis hominis* (n=36, 44.4%) but the least infection was seen in the workers of coffee shops (*P*=0.006) (Table 4).

**Table 4:** Prevalence of intestinal parasitic infections in eight occupational categories of 800 food handlers in Bandar Abbas, southern Iran, 2015

***Occupation***	***Frequency (%)***	***Parasitic infection (%)***
Bakery workers	81 (10.1)	44 (54.3)
Supermarket workers	150 (18.8)	52 (34.7)
Restaurant workers	171 (21.4)	58 (33.9)
Fast food workers	62 (7.8)	22 (35.7)
Butchers	33 (4.1)	9 (27.3)
Coffee shop workers	86 (10.8)	23 (26.7)
Office servants	161 (20.1)	48 (29.8)
Factory workers	56 (7)	23 (41.1)
Total	800 (100)	279 (34.9)

Living at work place and direct contact with raw foodstuff, affected the prevalence of intestinal parasites (*P*=0.000, 0.038 respectively) but the number of the family members and that of colleagues showed no effect (*P*=0.754, 0.582 respectively). Among the office servants including butlers, chef, and cleaners, the butlers had low contamination but the cleaners had the most (*P*=0.000).

## Discussion

In this study, 34.9% of participants were positive for intestinal parasites. Comparing to the other studies the results of the present study showed that parasitic infection in this area marked with the higher prevalence. Since the prevalence of intestinal parasites in a community is different according to the geographical and climate condition of the area, nutrition culture, poverty, malnutrition, personal and community hygiene and population density, therefore, the prevalence of intestinal parasites is different in some studies in Iran and other countries. The high prevalence of intestinal parasites in our study was in agreement with the findings of other studies, i.e., 29.1% in a study carried out in northwest Ethiopia (5), 29.3%, in northeastern of India (9), 29.4% in Khartoum, Sudan (11) and in disagreement with a study in north India (1.3%–7%) (12). The prevalence of intestinal parasites in food handlers of Khorramabad, western Iran (6) Sari, northern Iran (1) and Shiraz, central Iran (4) and Kerman in southeast Iran (8) marked 9%, 15.5%, 10.4% and 14.7% respectively. Bandar Abbas has warm climate with high humidity but low rainfall, especially in recent years, and because of low soil moisture, we did not expect high infection in intestinal parasites, neither pathogenic nor nonpathogenic.

Despite the majority of participants (90.1%) owned the health certificate, it indicates the state of poor sanitation. An irregularity in the performance of strict sanitary and lack of adequate monitoring is shown since there was not any statistically significant difference between owning health certificate and the risk of infection. We were also seeking to find out the rate of infection in food handlers to be much lower than that prevalence (13) in the population. According to existing standards, staff of food supply units such as bakery, confectionery, restaurant workers are screened for parasitological infections twice in a year and treated if necessary and in such occupations as dried goods seller, supermarkets, butchers, chicken shops that are less likely to transmit the parasitic infection, the number of screening tests is reduced to once per year (4).

Although the least infection was seen in the academic category, there was not any statistically significant difference between education level and the risk of infection that is in agreement with a study (14) and disagreement with other studies (15, 16).

No statistically significant difference between the prevalence of intestinal parasites found among the people lived in rural or urban areas similar to an investigation carried out in northern Iran (1).

Unlike some investigations (4), there was a statistically significant difference between occupation category and intestinal parasites, in a way that, the highest infection were seen in bakery workers.

Although no significant difference was observed among the number of colleagues and intestinal parasites, it seems to be relevant variable in bakeries because the majority of them live together at workplace.

The infection with more than one parasite was seen in the supermarket and restaurant workers in such a way that triple or quadruple infection was seen in the supermarket workers especially those in contact with fruits and vegetable, this is in accordance with the study that was carried out in northern Iran (1). The high prevalence of protozoan parasites in our study was consistent with the findings of the studies in other parts of the country and also other countries (1, 4, 6, 17) while a high prevalence of soil-transmitted helminths were seen in Indonesia (15) and southwest Ethiopia (18). The prevalence of intestinal parasitic among the food handlers in these regions was 83.9% and 44.1% and *A. lumbricoides* with 26.8% and 17.8% were the predominant parasites respectively. In the past decade, there seems to have been a general downward trend in the incidence of intestinal helminthiasis in Iran (19). Low prevalence of helminthic parasites in comparison with protozoan parasites in our study often depends on the transmission route. The low soil moisture, limited vegetation, supplied drinking water, hot sunlight, not using human waste as fertilizer and improvement in environmental health such as asphalt of the streets or alleys are the reasons for this condition. The high prevalence of protozoan parasites in participants who were in direct contact with food and the individuals living at workplace is the evidence for the simple route of transmitting of protozoa. Furthermore, the high proliferation of protozoa and the ability to produce cysts and resistance against environmental conditions are the reasons which explain this situation.

The most common parasite in our study, *B. hominis* (24.3%) was in agreement with the study (22.1%) (20). Since this parasite is not reported or detected in most clinical laboratories, its prevalence probably is higher than is recorded in some studies. The results of this study also showed that among infected persons with *B. hominis,* diarrhea was seen when accompanied with *G. lamblia* or *Dientamoeba fragilis*. Probably other protozoa may cause diarrhea in those people other than *B.hominis.* But since *B. hominis* is a zoonotic parasite and has a potential of causing disease, it is important to pay more attention where more research is required to be done regarding it (7).

*E. histolytica/dispar* was recorded only in 0.9% of participants but the most abundant parasite was *E. histolytica/dispar* (70.8% and 36.6% respectively) (21, 22).

*G. lamblia* was detected in 6.8% of the examined samples. This finding is inconsistent with the reports on the prevalence of the parasite in Sudan (20.5%) (23), Anatolia (26.8%) (24) and Ethiopia (0.8% and 18.8%) (5, 21) and consistent with the studies where carried out in Khuzestan, southwest Iran (4.52%) (25), and Gorgan, northern Iran (4.3%) (26).

Unlike the other studies (6, 8, 21) *D. fragilis* was seen in 4.3% of the subjects. Laboratory diagnosis of this parasite with routine methods such as wet mount and formalin-ether concentration is very difficult so permanent staining of the stool samples detected this parasite, hence, studies that did not use this procedure show the low prevalence of this parasite. Since this parasite is a pathogen and its route of transmitting is not exactly clear, other routes of transmitting should be considered.

There are different reports on the prevalence of intestinal parasites in men and women, but in our study, similar to other studies (6) no statistically significant difference found between the gender and intestinal parasites unlike the study (1, 14).

No statistically significant difference between the prevalence of intestinal parasites found among each age group similar to the investigation conducted in Khorramabad, western Iran (6), Kerman, southeast Iran (8) and Jakarta in Indonesia (15). It probably represents an equal exposure to the infective materials.

Although the number of family members seems to be effective in the prevalence of intestinal parasites, no significant difference was observed in this study unlike the study in Sari, northern Iran (1). A specific diagnostic method used for parasites such as *E. vermicularis*, unfortunately, which was not possible due to lack of cooperation of the participants.

## Conclusion

Although the prevalence of parasitic helminths, especially soil-transmitted helminths, is much reduced, the high prevalence of parasitic protozoa is still important issue in food handlers. Therefore, the control and prevention of intestinal parasites must be carefully monitored every six months, especially for those involved in direct contact with raw foods.

Routine methods alone are not enough for the detection of intestinal parasites so the concentration procedures must be done. In addition, the parasite workshops and its transmission should be held where workers are obliged to participate.

## Ethical considerations

Ethical issues (Including plagiarism, informed consent, misconduct, data fabrication and/or falsification, double publication and/or submission, redundancy, etc.) have been completely observed by the authors.
